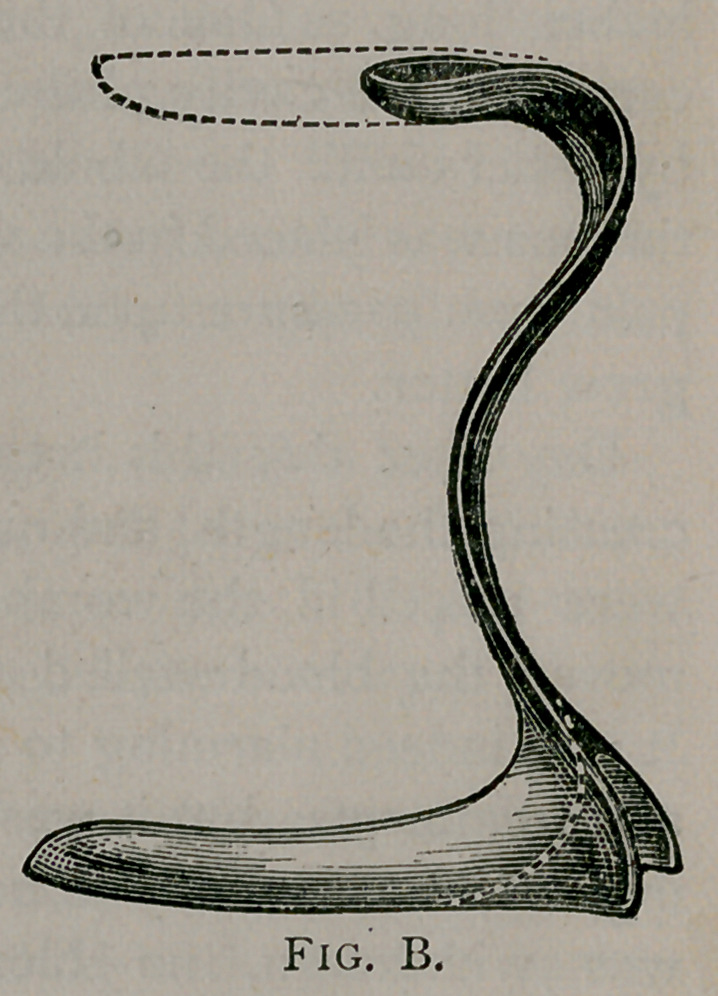# The Application of Intra-Uterine Pressure by the Tampon in Diseases of the Endometrium

**Published:** 1886-10

**Authors:** V. H. Taliaferro

**Affiliations:** Professor of Obstetrics and Diseases of Women and Children in the Atlanta Medical College, Atlanta, Georgia


THE ATLANTA MEDICAL AND SURGICAL JOURNAL.

VOL. III. OCTOBER, 1886. No. 8.

### Original Communications.

### THE APPLICATION OF INTRA-UTERINE PRESSURE BY THE TAMPON IN DISEASES OF THE ENDOMETRIUM.

BY V. H. TALIAFERRO, M. D., PROFESSOR OF OBSTETRICS AND DISEASES OF WOMEN AND CHILDREN IN THE ATLANTA MEDICAL COLLEGE, ATLANTA, GEORGIA.

Becoming convinced more than ten years ago of the damaging sequelæ of caustics to the uterine cavity, it has been my constant study to cure the diseases of that structure without them. It was in this line of study that I was led to the "application of pressure in diseases of the uterus, ovaries and peri-uterine structures," as published in the Transactions of the Medical Association of Georgia in April, 1878. It will be remembered that in September, 1882, in THE ATLANTA MEDICAL AND SURGICAL JOURNAL, I published my second paper upon this subject. In that paper I felt it my duty to take to task Dr. Nathan Bozeman, of New York, for his claim to priority of my use of the tampon in a paper read by himself at the meeting of the American Gynecological Society, September, 1878, five months subsequent to the publication of my first paper, which he had read and to which he made no allusion. Dr. Bozeman replied to my strictures in a series of articles running through several numbers of THE ATLANTA MEDICAL AND SURGICAL JOURNAL, with many illustrations. These profuse illustrations embodied nearly all the bungling and cranky appliances peculiar to Dr. Bozeman, none of which, so far as I know, are in use by the profession. If there is a gynecologist in this or in any other country who has adopted these instruments, he has not made it known. Dr. Bozeman gives illustrations of my speculum and his own to show by comparison that I got the idea from him. Unless blinded by egotism the doctor must know better. He must know, as I stated, that I got the idea from Sims, where he got his. The Sims speculum marks an epoch in gynecology, and the many varieties of perineal retractors in use since are but modifications of Sims'. I claim no originality for my speculum other than in its *modification* from the original Sims, and were I to claim more I should feel myself guilty of unpardonable literary theft. The only use I have, or have ever had, for Dr. Bozeman's speculæ, as well as his other clumsy appliances, is to exhibit them to my classes at the Atlanta Medical College as cranky and worthless curiosities; as such they must inevitably pass to history.

The effort of Dr. Bozeman to appropriate my own work is but a repetition of his old tricks, as will appear from the following quotation from an address by the illustrious and sainted Sims, delivered before the New York Academy of Medicine, on the 18th of November, 1857, upon the subject of *Silver Sutures in Surgery*. After detailing his laborious trials and failures with vesico-vaginal fistula, and his final success with the silver suture, on the 21st of June, 1849, he says: "The city of Montgomery, Alabama, was the theatre of my early operations. Bad health compelled me to leave there in 1853. I then gave Dr. Bozeman, of that place, a partnership in business, and indoctrinated him in my peculiar method of operating for vesico-vaginal fistula, instructing him in my various modes of using silver wire as a suture, not only in this class of affections, but in general surgery. Not understanding its principles of action, and therefore failing in its practical application, he was quite disheartened with his ill success, when by mere accident he fell upon a plan of fastening the wire and so modifying my method that in awkward or inexperienced hands it became easier of application. . . . . . Notwithstanding the fact that the doctor lived in Montgomery for years, without professional position till I gave it to him, that he is indebted to me for what he could never have obtained without my aid, he appropriates to himself every step of the operation that resulted from my own individual and unaided efforts—*even my silver wire and perforated shot*, the only things of any real *value whatever*, and publishes it as his operation 'by a new mode of suture,' making strenuous efforts to place my labors entirely in the back-ground.

"I do not complain of modifications, but I do complain of a disingenuousness that would be dishonorable under widely different circumstances.

"While I know that posterity will do me full justice, I do not even fear the verdict of my contemporaries, where the whole of the facts and their philosophy are laid plainly before a just and discriminating profession. But, sir, if you wish to offer a premium for the encouragement of secret remedies, rob me of my well-earned claims as a discoverer and propagator of a great principle that shall live as long as surgery is cultivated as a science or practiced as an art; and if you are particularly desirous to drive your young men, ambitious of honorable distinction, from their high resolves to elevate and ennoble our calling, show them that you are ever ready to thus endorse any attempt to detract from the meed of their self-sacrificing efforts."

I have thus quoted somewhat at length from Sims' address before the New York Academy of Medicine, published by order of the Academy in 1858. Sims lived after this to build up the field of gynecology and to make it what it is. His name and his memory will live in grateful remembrance through the ages, while his detractors and purloiners must occupy the dark and forgotten pages in history. Sims lived to establish himself in the hearts of every civilized nation on the earth. Above all, he leaves as an example for humanity a *stainless Christian character*. Being without envy, malice or jealousy himself, he never suspected, nor could he understand them in others. We can well imagine the pain and suffering, in one of his sensitive nature, caused by bold and dishonorable effort to pluck his honors—honors achieved by the most laborious and self-sacrificing labor. In the remarkable instance cited it was not only the attempt to wear the honors of another, but the effort to sacrifice the trusting, confiding friend.

Sims was incapable of untruth or injustice. He continually labored to build up others. His generosity was as unbounded as his genius. The egotism and selfishness which could so blind the better elements of human nature as to lead to the gross ingratitude which Sims depicts deserves, as it must reap, eternal condemnation.

I have made these quotations from Dr. Sims and drawn my deductions from them in order to show the character of the man who claims the reward of my devoted and earnest labor. Upon what ground, forsooth, does this man lay his claim? Does he point to priority in publication? No. Does he produce reliable, living testimony to his claim? No. Upon what, then, does he base priority? Nothing—absolutely nothing—as in the case of Sims (as above quoted), but his own bare statements, and the older members of the profession know how much this is worth.

I have used hard words, and the subject justifies it. Soft words don't meet cases like this. The younger members of the profession should be made to feel the enormity of literary theft. To steal the brain-work of a co-laborer, next to appropriating the work of a *friend* and co-laborer, is the meanest act on the earth. From big men we expect big things. The higher in professional scale the more culpable such foul work. Enough of this man so long besmirched by the odium of professional obloquy.

At the late meeting of the Medical Association of Georgia, I read a paper upon the "Application of Pressure by the Tampon to the Cavity of the Uterus for Diseases of the Endometrium." Since this publication there appeared in THE ATLANTA MEDICAL AND SURGICAL JOURNAL a translation from the French, by Dr. V. O. Hardon, of this city, upon "Vulliet's New Method of Dilating the Uterine Cavity." It does not seem to have occurred to Vulliet that his method of dilatation was a valuable *therapeutic resource.* Indeed, the pressure used for dilatation so rapidly modifies the diseased structure it is designed to diagnose that its value as a means of diagnosis is greatly impaired. The more rapid methods of dilatation are far better than by the slow process of the tampon. By the time the dilatation is sufficient for visual exploration, the endometritis, mucous fungosities, polypoid growths, etc., that may have existed will often have disappeared entirely. As a means of dilatation of the uterine cavity the tampon is valuable, but as a means of *diagnosis* it is well-nigh valueless, except in fibroid growths, which from their density would be less affected by the pressure. In such cases as this the touch is far more reliable than the eye, and a tent that would dilate rapidily and admit the finger would afford far better opportunity of correct diagnosis.

As a therapeutic measure, the *intra-uterine* tampon is simple, safe and marvelous. Chronic inflammation, granular erosions, mucous fungosities, together with the usual subinvolution, rapidly disappear under the influence of the pressure. With the proper observance of the contra-indications, to all intra-uterine applications, the remedy is perfectly safe. It should, of course, never be used when there is the vestige of inflammation in the peri-uterine structures. I have now a patient at my private infirmary whose uterus has been packed from the fundus to the end of the cervix for more than a month, who rarely goes to bed, except by my instructions, for some hours immediately following the application. So long as there is sufficient oozing of blood to saturate the tampon, it is removed and re-applied daily; otherwise it remains for two days. If iodoform is used with the packing, as should always be done, the patient is secure against sepsis, the dressing removed being clean and free from odor, though it remain for days. I not unfrequently leave it alone for three days.

The illustrations convey quite a correct idea of the method of applying the tampon. The patient is placed in the knee-chest position, or Sims' semi-prone, and the perineum retracted with a Sims speculum, or with any perineal retractor. I use my own speculum, as it separates the vulva and vaginal orifice better, and gives a more satisfactory command of the parts. The anterior lip of the cervix is seized with a tenaculum and the uterus lifted forward toward the vaginal outlet. A little pledget or roll of cotton, with a thread wound about it, is now seized with the dressing forceps (with small blades) and carried in the uterus quite to the fundus; this being done the blades are sufficiently separated to loosen their hold and partially removed, and the cotton roll caught in a new place and carried up, as was the first, the maneuver being repeated again and again until the little roll is thoroughly packed away in the uterus. These little cotton rolls are repeated one on another until the entire cavity is filled. Each piece of cotton is wound about (as represented in the diagram) with strong spool thread with a long end left to better facilitate and insure removal. The size and length of the cotton roll should correspond with size and depth of cavity to be filled. I have packed as much as one and three-quarter yards of lamp-wick in a single piece in the cavity of the body. The cavity in this case was unusually large and filled with granulations. I tried the lamp-wick because of its convenience, but discarded it on account of its hardness and want of elasticity. Nothing does so well as *new clean cotton.* The new cotton does not need washing or other preparation. Absorbent cotton does not do so well—it packs too hard and is inelastic. I usually get clean sample cotton from the cotton factors. In this way we can feel sure of using an article that has never been in use. *

The following cases, treated conjointly by my associate, Dr. Noble, and myself, are selected because of their usually intracta-

* In *the beginning* of the treatment in ordinary endometritis the tampon should not be carried further than just within the internal os—the cavity being gradually and carefully encroached upon until the fundus is reached. When the uterus is filled with fungus granulations the tampon should be carried at once to the fundus. The intra-uterine tampon should always be accompanied by a small but firm vaginal tampon—using glycerine and iodoform on the first pledgets. The patient should be kept in bed for some hours following the treatment, or if there be pain, so long as this continues. If pain is persistent the tampon should be removed.

LEO ROCK ENGR.
ATLANTA GA.

ble nature and the slow and unsatisfactory results of the ordinary methods of treatment. Dr. Noble has been kind enough to write out these cases for me, and I give them just as prepared, vouching for their accuracy in every detail. It will be seen that the first case dates back more than two years ago. Since this time Dr. Noble and myself have treated many cases by this method, and I give it now to the profession for the first time in detail, with the assurance of its safety and inestimable value:

In 1871, and while living at Columbus, Ga., I published a paper upon "Uterine *cloth tents* in diseases of the body and cavity of the uterus." In that paper the following language is used: "For some years previous to the adoption of the cloth tent I had used strips of cloth or linen saturated with Churchill's tincture of iodine, solution of carbolic acid, or other substances to be used. One end of the strip, folded over the point of a small probe, was passed to the fundus of the uterus; the probe being withdrawn, was again and again introduced, carrying with it at each time an additional fold of the cloth until the cavity was completely *packed*. This *packing* was permitted to remain until expelled by the contractions of the uterus, which required usually from six to twelve hours. The firmer the packing the more active the contractions and the quicker its expulsion. It was to avoid unnecessary contractile action and to make the strips self-retaining that led to the idea of rolling the cloth or linen strip into a tent, and thus the better to control, by its size and length, the contractions of the uterus, and to secure its retention for any desirable length of time."

Thus it will be seen that several years before the publication of this paper, in 1871, I was in the habit of *packing* the uterine cavity. I did not then know the value of the *pressure* I had secured by the intra-uterine tampon, the object then being the thorough medication it afforded. Thus, for the time being, slipped from my hands a remedy of marvelous power.

It will be observed that the intra-uterine packings were usually expelled by the contractions of the uterus induced by its pressure. I have since learned that this occurred then, as it does now, in a certain class of cases: where there is simply mucus inflammation, with more or less sensitiveness. Where there is a large patulous cavity none occurs, nor does it occur in any case if the uterine cavity is slowly and gradually encroached upon and tolerance induced.

CASES.

*Case 1.*—This case is reported because it was the first upon which we used the intra-uterine tampon. The patient was large, obese and anæmic—almost completely exsanguinated. Her health had been good up to her last confinement, two years prior to the commencement of her treatment.* In that labor the cervix sustained a laceration on either side to the vaginal junction.

She had the usual train of symptoms dependent upon such a lesion and upon sub-involution. Six months after labor she began to have hemorrhage from the uterus, which continued almost constantly for eighteen months. There was vesical tenesmus. The bowels were constipated, digestion greatly impaired and strength gone. She was confined to bed from sheer exhaustion.

The perineum was partially lacerated. The uterus was about the size of a three months' pregnancy and prolapsed to the floor of the pelvis. There was great eversion of both the anterior and posterior lips, with granular degeneration or fungosities of the endometrium. The cervical canal and internal os were quite patulous, which permitted thorough exploration. A slight touch was sufficient to produce a hemorrhage, serious to a person in her condition.

The treatment consisted in the proper regulation of diet, nourishing food, tonics, etc., with daily iodoform and glycerine dressings, in the vagina, of sufficient firmness to control the hemorrhage.

In four days she recovered strength enough to sit in an easy chair the greater part of the day, and at the end of a week walked from the house to the street. Seven days later she walked one-third of a mile. At this time, the end of the second week, the bleeding was so free when the tampon was removed from the

*Treatment began in February, 1884.

vagina that it was useless to attempt to keep it sponged away. It was therefore decided to pack the cavity to stop it.* This we did daily, as described by Dr. Taliaferro himself.

The improvement was marked as it progressed by a gradual decrease in the bleeding at each treatment, and in a few days by the appearance of pus, which gradually increased as the packing went on. Immediately following the removal of first few dressings there was very free bleeding, but this was of no moment, as it was quickly checked by the fresh packing.

We continued this treatment for eight weeks, at the end of which time there was not even a stain of blood upon the cotton as it came away. We subsequently found to our astonishment that we came near producing atrophy of the uterus, as that organ was just a little under the normal size for a multipara.

*Case 2.*—I report this case because of its most unfavorable condition for such treatment. In fact it was contra-indicated. The patient was a small Irish woman with a history of good health up to two years prior to the time I first saw her. Her trouble began with a miscarriage, complicated with bilateral laceration of the cervix and post-partem hemorrhage. She said the placenta was removed by the hand. The usual train of symptoms accompanying this laceration and sub-involution followed with occasionally an attack of peri-uterine inflammation. She had metrorrhagia for two months before I saw her, for which she was treated by another physician with tents. This resulted in peri-uterine inflammation and a very great increase of the bleeding. Tamponing the vagina was required to check it.

Though active inflammation had been going on, she bled until she was reduced to a sub-normal temperature when I first saw her. At the end of two weeks the inflammation had subsided, and an exploration of the pelvic organs was made. The uterus was nearly as large as an infant's head, almost filling the pelvic cavity. Some inflammatory deposits were in the broad ligaments, yet the womb was slightly movable. The cervical portion of

*"Just after the war" Dr. Taliaferro made a practice of packing the uterus with strips of cloth with a view to medication, but found that in a few hours they were invariably forced out. In the summer of 1882, I successfully dilated the uterus with cotton tampon for the removal of a fibroid polypus, three inches in diameter, without having the packing thrown out.

the os was three inches in diameter and very greatly congested. A slight brush would remove the epithelium and set up a profuse oozing of blood from the cervico-vaginal mucous membrane. The os and cavity was granular. The cervical canal and internal os were very much dilated. The lips were very much enlarged. The anterior lip was about the size of a black walnut and felt like a fibroid induration. During my absence from the State she had a recurrence of her menorrhagia, and I returned in time to check it just as it had increased to a hemorrhage that bled her almost to syncope while lying in bed. Her circulation was in a very feeble condition from the loss of blood.

I began the uterine packing at once, as she had no more blood to spare, guarding it with a vaginal tampon. Each treatment was followed by a decrease of blood and a corresponding improvement in strength. At first the uterine cavity seemed to dilate until I could pack three little soft twists of absorbent cotton about half the size of a lead pencil and about fourteen inches long into it. Then it began to give her pain—a bearing-down pain—for about two hours after its introduction. As the improvement went on the uterus contracted more firmly until finally it threw the whole dressing into the vagina. The blood had ceased to stain the dressing for three weeks, and as other evidences of disease had disappeared, I decided to leave the treatment off for a few days that I might determine the size of the uterus. This I found was normal, and as she refused an operation on the cervix, the case was dismissed, at the end of the fifth week, cured—all but the cervical rupture.

This case is interesting from the fact that such a large subinvoluted uterus was cured in five weeks, and from the fact that this treatment followed without detriment an attack of peri-uterine inflammation, while there yet remained inflammatory deposits in the pelvis.

*Case 3.*—This case is an inmate of our infirmary and still under treatment. She is a large and corpulent woman, very anæmic, has been unwell since her last child-birth, thirteen years ago, and bleeding from the uterus for five years almost constantly. Slight exertion caused a great increase in the flow; she was therefore confined to the bed a great part of the time. She gave no history of peri-uterine inflammation. The uterus was sub-involuted, and fully as large as a three-month pregnancy. There were large fungus granulations throughout the cavity extending to the external os. Deep lacerations of the cervix extended on both sides to the vaginal junction. There was great distress and soreness in the rectum, due to the presence of a hard ball of fœcal matter. This mass was about the size of an orange, somewhat oblong and pointed at the lower extremity. For thirteen months it had been presenting at the anus during defecation, receding to the sigmoid flexure when the bowel was inactive. The act of defecation caused great pain and distressing tenesmus.

A surgeon of acknowledged reputation visited her at her home in a distant portion of this State, and as she states made a diagnosis of fibroid tumor of the uterus and stretched the sphincter ani. The day of her entrance to our infirmary, I pressed the mass down in the pelvis with one hand from above the brim, and by the aid of my finger and ox-gall enemetas completely cleared the rectum. This put an immediate end to the painful defecation. The following day we began the intra-uterine packing with little rolls of cotton about half the size of a lead pencil and about fifteen inches long. One of these was passed through the patulous canal into the cavity of the uterus to the fundus, and this followed by others until the whole organ was filled up, and then a firm tampon was placed in the vaginal vault. This latter caused some pain from pressure upon the sore rectum, but the soreness rapidly grew better.

Day after day this treatment was carried out, gradually increasing the length and number of the little cotton twists that were placed in the womb. As the first few dressings were removed the blood welled up from the uterus like a little spring. It was indeed alarming to persons not accustomed to such sights and treatments, but it was promptly checked by the fresh packing, which was ready to be introduced as soon as the old one was withdrawn, no blood of any consequence was lost. This was repeated at each treatment, but in a less degree, until the end of the third week, when there was only a stain of blood on the dressing.

At this time the length of the packing-twist was one and three-fourth yards. All this was carried beyond the internal os, and about the same quantity or more was packed in the cervix.

At the present writing there is some stain of blood as the dressings are withdrawn, but nothing like a bleeding ever occurs. There is quite a profuse coating of pus upon the cotton when taken away. The cessation of the hemorrhage and the appearance of pus show a destruction of the granulations, but the packing is continued for farther reduction of the sub-involution.

The uterus at first seemed to dilate, but now, the fifth week, it is regaining its proper tone and vigor; it seems to contract, and is reducing both in volume and calibre of its cavity, and we now have to introduce smaller packings.

These cases are reported rather briefly, and many details of the general treatment left out, as it was the effect of the packing that we wish to note. The first case was treated at the office, the second at her residence, and the third is an inmate of our infirmary.

FIG. A.

FIG. B.

These are but selections of quite a number that have been and are now under treatment.

As Dr. Bozeman claims that our speculum is an infringement on his, it has been thought best to set the cuts of them both side by side that the contrast between them may be observed.

Fig. A represents Bozeman's instrument with its flat blade and cutting edge. Fig. B represents Dr. Taliaferro's speculum with its thin, somewhat flattened blade and smooth, rounded edges. The dotted lines at the bottom show where we have added the flanges to the angle of Sims' speculum, and those at the top show that the upper blade has been cut away and a sort of handle made of the stub that was left.

It is plainly seen that both instruments are simply modifications of Sims'.

## Figures and Tables

**Figure f1:**
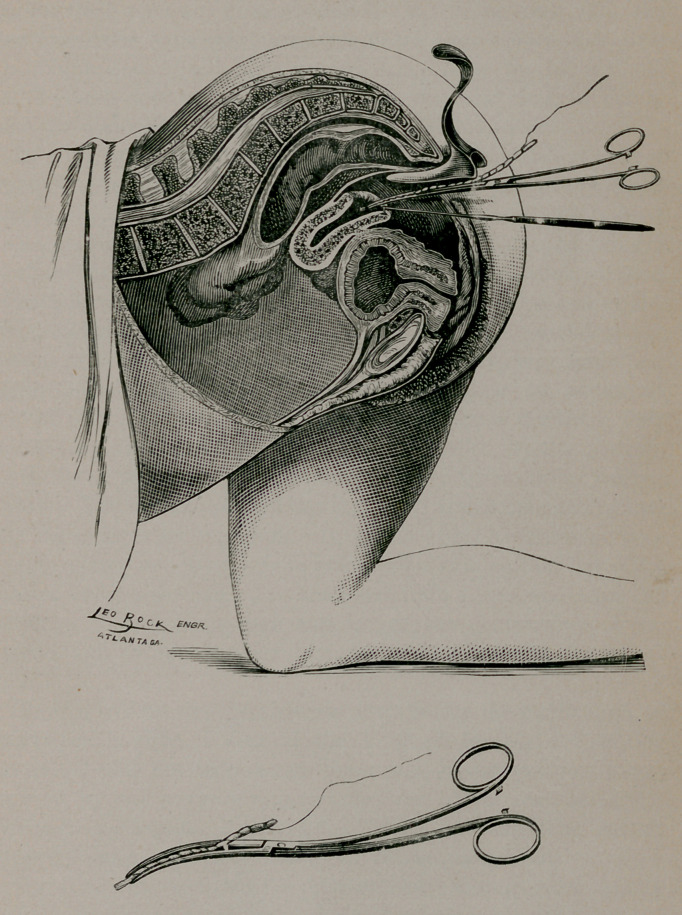


**Fig. A. f2:**
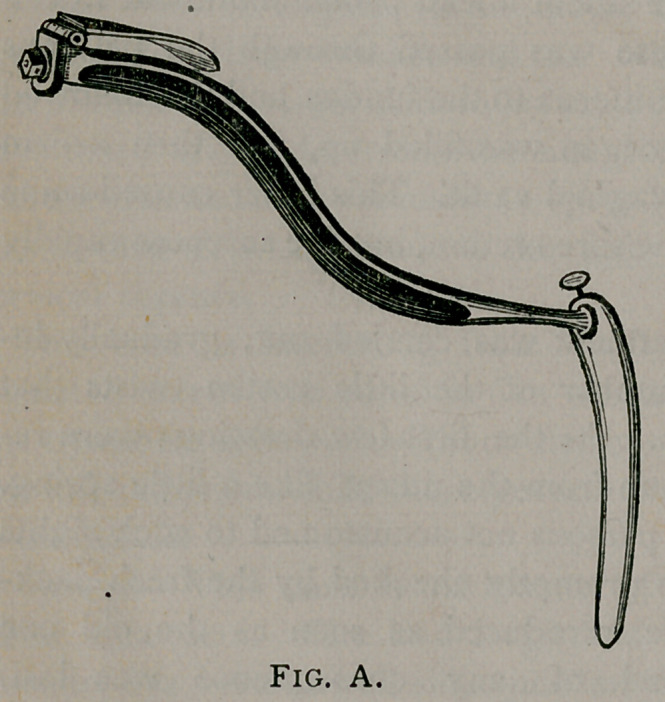


**Fig. B. f3:**